# Experimental hookworm infection: a randomized placebo-controlled trial in asthma

**DOI:** 10.1111/j.1365-2222.2009.03433.x

**Published:** 2010-02

**Authors:** J R Feary, A J Venn, K Mortimer, A P Brown, D Hooi, F H Falcone, D I Pritchard, J R Britton

**Affiliations:** 1 Division of Epidemiology and Public Health, University of NottinghamNottingham, UK; 2 Division of Respiratory Medicine, University of NottinghamNottingham, UK; 3 School of Pharmacy, University of NottinghamNottingham, UK

**Keywords:** airway responsiveness, asthma, helminth, hookworm, intervention, *Necator americanus*, randomized-controlled trial

## Abstract

**Background:**

Epidemiological studies suggest that hookworm infection protects against asthma, and therefore that hookworm infection may have a direct or an indirect therapeutic potential in this disease. We now report the first clinical trial of experimental hookworm infection in people with allergic asthma.

**Objectives:**

To determine the effects of experimental hookworm infection in asthma.

**Methods:**

Thirty-two individuals with asthma and measurable airway responsiveness to adenosine monophosphate (AMP) were randomized and double blinded to cutaneous administration of either ten *Necator americanus* larvae, or histamine solution (placebo), and followed for 16 weeks. The primary outcome was the change in provocation dose of inhaled AMP required to reduce forced expiratory volume in 1 s by 20% (PD_20_AMP) from baseline to week 16. Secondary outcomes included change in several measures of asthma control and allergen skin sensitivity and the occurrence of adverse effects.

**Results:**

Mean PD_20_AMP improved in both groups, more in the hookworm [1.49 doubling doses (DD)] than the placebo group (0.98 DD), but the difference between groups was not significant (0.51 DD; 95% confidence interval: −1.79 to 2.80; *P*=0.65). There were no significant differences between the two groups for other measures of asthma control or allergen skin sensitization. Infection was generally well tolerated.

**Conclusions:**

Experimental infection with ten hookworm larvae in asthma did not result in significant improvement in bronchial responsiveness or other measures of asthma control in this study. However, infection was well tolerated and resulted in a non-significant improvement in airway responsiveness, indicating that further studies that mimic more closely natural infection are feasible and should be undertaken.

*Cite this as*: J. R. Feary, A. J. Venn, K. Mortimer, A. P Brown, D. Hooi, F. H. Falcone, D. I. Pritchard and J. R. Britton, *Clinical & Experimental Allergy*, 2010 (40) 299– 306.

## Introduction

Asthma is one of the commonest chronic diseases of developed countries, but despite significant investment, research into the aetiology of asthma has yet to identify the major avoidable causes, and the available treatments remain poorly effective in around 10% of patients [1]. New approaches to the prevention and treatment of asthma are urgently needed. There is now substantial epidemiological evidence that intestinal parasite infection in general, and hookworm infection in particular, may protect against wheeze, asthma and other allergic disease [2, 3]. These findings raise the possibility that experimentally induced parasite infections may be able to contribute, directly or indirectly, to the development of new strategies for the management of asthma.

We have previously reported two studies carried out in preparation for clinical trials of the effect of experimental hookworm infection in asthma. The first was a dose-ranging study in normal volunteers [4], which found that infection with ten *Necator americanus* larvae established a level of intensity of infection (>50 eggs/g feces), as identified in our previous epidemiological work, to be strongly associated with protection against asthma symptoms [5]. The second was a safety study in people with allergic rhinoconjunctivitis and measurable airway responsiveness but not clinically diagnosed asthma, which established that the lung migration phase of experimental hookworm infection was well tolerated and did not significantly exacerbate airway responsiveness [6]. This study also showed that an immunological phenotype was induced, which corresponded in many ways to that seen in natural infection [7], supporting the use of iatrogenic infection in a therapeutic context. We now report the first randomized double-blind placebo-controlled study of the effects of experimental hookworm infection in people with asthma.

## Methods

### Participants

Potential participants aged 18 years and over with a physician's diagnosis of asthma treated with daily inhaled corticosteroids of up to 1000 mcg beclometasone (or equivalent) were identified by local advertisement, sent an information sheet and invited to attend a screening visit.

At the screening visit, after excluding those with significant medical problems (other than asthma) and women who were pregnant or pre-menopausal and unwilling to use contraception for the duration of the study, written informed consent was requested from all participants. In those who consented, lung function was measured according to international guidelines [8] using a Spirometer (Vitalograph, Buckingham, UK) taking the forced expiratory volume in 1 s (FEV_1_) as the higher of two values within 100 mL. Bronchial hyperresponsiveness (BHR) was measured by adenosine monophosphate (AMP) challenge giving sequential inhalations in doubling increments from 0.115 to 944 μm at 2 min intervals until FEV_1_ fell by at least 20% from post-saline baseline or until the maximum dose of AMP had been inhaled, as described previously [6]. The AMP dose required to reduce FEV_1_ by 20% (PD_20_AMP) was estimated by an interpolation between the two last doses on the log dose-response plot [8]. Venous blood was collected for haemoglobin estimation, differential white cell counting and serum albumin. Allergen skin sensitization to *Dermatophagoides pteronyssinus*, cat fur, grass pollen and positive (histamine) and negative (saline) controls (Diagenics Ltd, Milton Keynes, UK) was measured using standard skin prick test methods defining a positive result as a mean weal diameter 3 mm greater than the negative control [9]. Urine analysis for β-HCG (QuickVue, Quidel Corporation, San Diego, CA, USA) was used to confirm that female participants were not pregnant. An interviewer-administered Juniper Asthma Quality of Life Questionnaire (AQLQ) based on participants' experiences over the preceding fortnight was completed [10]. Participants were not eligible if their FEV_1_ fell by <20% during the bronchial challenge, haemoglobin was below normal reference levels or they did not have a positive skin test response to at least one allergen.

All eligible participants had their inhaler technique reviewed and were provided with a miniWright peak flow meter (EU scale). Instruction on peak expiratory flow (PEF) measurement was given and inhaler technique was optimized. For the duration of the study, participants were asked to continue their usual asthma medication and to use short-acting inhaled β_2_-agonists for a relief of asthma symptoms.

### Randomization

Participants were seen after a 2-week run-in period for concealed randomization to active or placebo infection, allocated in blocks of four according to a computer-generated random code. Larvae were obtained by a culture of fecal material from a healthy human source known to be negative for hepatitis B and C and human immunodeficiency virus, as described previously [11]. Two hundred microlitres of water containing ten *N. americanus* larvae (active infection) or 200 μL histamine dihydrochloride solution (1.7 mg/mL) (placebo) was then administered to an area of skin on the forearm and covered with gauze and water-tight adhesive dressing for 24 h. To ensure the clinical researcher carrying out the protocol measures remained blind to treatment allocation, infection solutions were administered by an independent member of the research team not involved in the study measurements.

### Follow-up

After randomization, participants attended study visits every fortnight for 8 weeks, and at 12 and 16 weeks. Our original protocol specified 12 weeks follow-up, but because the eosinophilia we observed in the safety study was still decreasing at 12 weeks [6] before commencing the present study, we elected to increase follow-up to 16 weeks. At each study visit, the Juniper AQLQ was completed, venous blood was taken for the same tests as at the screening visit and a stool sample, collected within the preceding 24 h, was provided for quantification of egg burden, as described previously [4]. PD_20_AMP was measured, and if after enrolment in the study, participants no longer responded with a 20% reduction in FEV_1_ at below the maximum administered dose of AMP, a censored value of 1888 μM was assigned, which is one doubling dose higher than the maximum dose of AMP administered [12, 13]. An additional full blood count test was carried out 3 weeks after randomization to coincide with the expected time of onset of hookworm-associated eosinophilia [4, 6]. For the duration of the study, including the 2-week run-in period before randomization, participants completed a daily diary, recording twice-daily PEF (best of three attempts), twice-daily asthma symptom scores on a scale of 0 (no symptoms) to 5 (maximal symptoms), use of reliever medication and scores for a range of pre-determined symptoms potentially attributable to hookworm infection on a scale from 0 (none) to 10 (maximum possible severity). Allergen skin tests were repeated at the final visit (week 16) and participants were then unblinded by the researcher who administered the infection or placebo at their randomization visit. Those in the hookworm group were supplied with mebendazole 100 mg twice a day for 3 days to eradicate the infection; stool egg counts and blood eosinophil counts were checked fortnightly in those completing therapy until egg counts were zero and eosinophils had returned to within 0.2 × 10^9^/L of their screening value.

### Safety and Ethics

Data on adverse effects and haemoglobin, eosinophil and serum albumin levels were monitored regularly by the trial statistician and a data monitoring committee. The study was approved by the Nottingham Research Ethics Committee, the Research and Development Department at Nottingham University Hospitals NHS Trust and registered with the Clinical Trials register (reference NCT00469989).

### Data analysis

The primary outcome was the change in PD_20_AMP from baseline (mean of screening and randomization visit values) to the week 16 (final visit) value expressed in doubling doses. Secondary outcome measures of asthma control were determined for each 2-week period of the study and comprised PEF variability (two-lowest percent mean [14]), percentage of symptom-free days and nights (determined using the twice daily asthma-control symptoms scores), and percentage of reliever inhaler-free days and nights and Juniper AQLQ scores [10]. We plotted these variables over time to assess any trend, and then computed the difference between baseline (run-in period or for Juniper AQLQ, the mean of the screening and randomization visits) and the end of the study (weeks 15 and 16). We also computed area under the curve (AUC) (GraphPad Prism 5, GraphPad Software Inc., San Diego, CA, USA) using the period weeks 5–16 as an alternative summary variable for PD_20_AMP and each asthma control measure to ensure that we did not miss an effect if the measurements taken at the end of the study had been spurious. Change in allergen skin sensitization (mm) was calculated as the difference between the baseline and week-16 saline-adjusted results. Mean daily symptom scores for adverse effects potentially attributable to the hookworm infection were compared over the entire 16 weeks and also for a pre-determined ‘high-risk’ period chosen, observed as the period during which the symptoms were most likely to occur in our previous studies [4, 6].

The two groups were compared using the independent samples *t*-test for all variables with the exception of the adverse effects symptoms and AUC variables, which were not normally distributed, could not be transformed and were therefore compared using the Mann–Whitney *U*-test. As this was a relatively small trial, we explored whether adjusting for potential confounders (variables listed in Table 1) using multiple linear regression affected the results. We repeated the analyses using data from just the first 4 weeks after randomization to determine whether there was evidence of exacerbation of asthma during the phase of larval pulmonary migration.

**Table 1:** Baseline characteristics of participants completing the study

	Hookworm (*n*=16)	Placebo (*n*=16)
*Demographics*		
Gender		
Male	8 (50%)	9 (56%)
Female	8 (50%)	7 (44%)
Age (years)		
Mean (SD)	40.9 (10.7)	39.8 (15.2)
Body mass index (kg/m^2^)		
Median (IQR)	25.6 (23.5, 26.8)	26.9 (23.8, 29.6)
Deprivation score*		
Median (IQR)	15.6 (9.0, 28.3)	13.3 (7.0, 30.3)
Smoking status		
Never	10 (63%)	11 (69%)
Ever	6 (38%)	5 (31%)
*Asthma medication*		
Daily inhaled corticosteroid dose		
<500 mcg/day	10 (63%)	9 (56%)
>=500 mcg/day	6 (38%)	7 (44%)
Past oral steroid usage		
No	8 (50%)	4 (25%)
Yes – not in last 2 years	5 (31%)	8 (50%)
Yes – in last 2 years	3 (19%)	4 (25%)
Daily long-acting β_2_-agonist usage		
None	11 (69%)	9 (56%)
Some	5 (31%)	7 (44%)

* Using index of multiple deprivations [15].

IQR, interquartile range; SD, standard deviation.

Three sensitivity analyses for each outcome were also performed excluding participants who: (i) breached the protocol, (ii) had <75% of potentially available data complete for the outcome variable and (iii) were infected with hookworm but did not have a positive stool culture for larvae at week 16. All data were analysed using STATA 10 SE (STATA Corp., College station, TX, USA).

### Sample size calculation

Our pre-study sample size calculation revealed a sample size of 30 (15 in each group) would provide 80% power to detect one doubling dose difference in our primary outcome, change in PD_20_AMP, between hookworm and placebo groups assuming a standard deviation of approximately one doubling dose (DD).

## Results

**Fig. 1:**
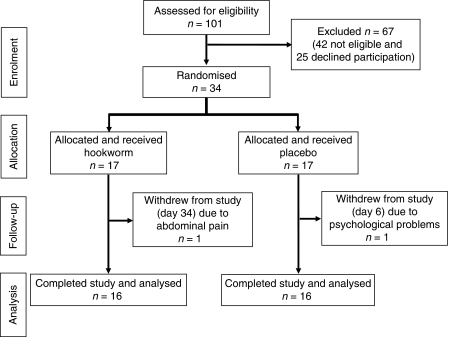
Flow chart of study participants.

Thirty-four participants were recruited between December 2006 and June 2007 with 17 allocated to each intervention. Two participants withdrew before our primary end-point could be measured, that is, before parasite maturation was complete (one from the placebo group on day 6 due to psychological problems, and one from the hookworm group on day 34 due to abdominal pain); they were excluded from further analysis (Fig. 1). Baseline characteristics of the two study groups were similar with respect to gender, age, body mass index, smoking history, index of multiple deprivation [15], use of asthma medication (Table 1), bronchial responsiveness (PD_20_AMP), lung function (percentage predicted FEV_1_ and PEF variability), Juniper AQLQ score and symptom-free and reliever-inhaler-free days and nights (Table 2).

**Table 2:** Baseline clinical measures

	Hookworm (*n*=16)	Placebo (*n*=16)
*Lung function [mean (SD)]*		
% predicted FEV_1_ at screening visit	83.38 (15.77)	87.06 (23.13)
% predicted FEV_1_ at randomization	80.25 (17.98)	85.94 (18.37)
*Bronchial responsiveness [median (IQR)]*		
PD_20_ AMP at screening visit	2.34 (0.78, 7.30)	3.76 (1.49, 7.52)*
PD_20_ AMP at randomization	4.10 (1.53, 11.26)†	6.98 (1.93, 17.22)
*Skin sensitization to allergen at the screening visit [weal size in mm; (mean (SD)]*		
Grass	5.13 (3.00)	5.31 (5.22)
Cat fur	4.25 (2.58)	3.91 (3.72)
*Dermatophagoides pteronyssinus*	6.53 (3.29)	6.38 (4.36)
*Juniper AQLQ scores*‡*[median (IQR)]*		
Juniper score at screening visit for run-in period	193.00 (186.00, 212.50)	175.00 (154.00, 203.00)
Juniper score at randomization for run-in period	200.00 (186.50, 212.00)	186.50 (174.00, 209.00)
*PEF variability*§*[median (IQR)]*		
PEF variability (%) during run-in period	91.99 (87.96, 93.10)	91.68 (85.62, 94.25)
*% symptom-free days/nights [median (IQR)]*		
% symptom-free days during run-in period	66.07 (21.43, 88.31)	46.43 (8.12, 96.43)
% symptom-free nights during run-in period	66.76 (40.66, 95.83)	85.71 (57.69, 92.58)
*% reliever-inhaler-free days/nights [median (IQR)]*		
% reliever-free days during run-in period	84.52 (7.42, 92.26)	60.71 (3.57, 92.86)
% reliever-free nights during run-in period	75.65 (44.78, 100.00)	92.86 (76.92, 100.00)

* *n*=13.

† *n*=15.

‡ Maxiumum score of 224 where the higher scores indicates better quality of life.

§ % PEF variability: a value of 100 indicates no variability, i.e. perfect control.

% predicted FEV1, percentage predicted forced expiratory volume in 1 s; AQLQ, Asthma Quality of Life Questionnaire; IQR, inter-quartile range; PD20AMP (DD), provocation dose of adenosine monophosphate to reduce forced expiratory volume in 1 s by 20% in doubling doses; PEF, peak expiratory flow; SD, standard deviation.

### Primary outcome

PD_20_AMP improved relative to baseline in 11 of the 16 participants finishing the study who received hookworm (69%) and in eight who received placebo (50%). Mean PD_20_AMP improved in the hookworm group by 1.49 (SD 2.00) DD and in the placebo group by 0.98 (4.02) DD, but the mean difference between these changes was not statistically significant [0.51 DD; 95% confidence interval (CI) −1.79 to 2.80; *P*=0.65, Table 3].

**Table 3:** Change in asthma and allergic outcomes from the baseline to the end of the study

	Mean (SD)				
	Hookworm (*n*=16)	Placebo (*n*=16)	Difference in means	95% CI	*P*-value*
*Bronchial responsiveness (DD)*					
Change in PD_20_AMP (DD) (final visit – mean of randomization and screening visits)	1.49 (2.00)	0.98 (4.02)	0.51	−1.79, 2.80	0.65
*Skin sensitivity (mm)*					
Change in skin sensitization to grass (final visit – screening visit)	−0.59 (2.42)	−0.19 (2.37)	−0.41	−2.14, 1.32	0.63
Change in skin sensitization to cat fur (final visit – screening visit)	0.19 (1.74)	0.03 (1.63)	0.16	−1.06, 1.37	0.79
Change in skin sensitization to *Dermatophagoides pteronyssinus* (final visit – screening visit)	−0.66 (1.46)	−0.19 (2.45)	−0.47	−1.94, 1.00	0.52
*Juniper AQLQ scores*†					
Change in Juniper score (final visit – mean of screening and randomization visits)	10.66 (14.08)	15.34 (22.41)	−4.69	−18.20, 8.82	0.48
*PEF variability*‡					
Change in PEF variability (%) (final 2 weeks – run-in period)	1.03 (6.17)	−1.21 (7.17)	2.23	−2.60, 7.06	0.35
*% symptom-free days/nights*					
Change in % symptom-free days (final 2 weeks – run-in period)	8.43 (39.67)	12.59 (46.39)	−4.16	−35.32, 27.01	0.79
Change in % symptom-free nights (final 2 weeks – run-in period)	9.05 (32.50)	12.72 (48.98)	−3.66	−33.68, 26.35	0.80
*% reliever free days/nights*					
Change in % reliever-free days (final 2 weeks – run-in period)	0.37 (24.87)	1.39 (34.46)	−1.03	−22.72, 20.67	0.92
Change in % reliever-free nights (final 2 weeks – run-in period)	−0.01 (21.49)	3.59 (27.17)	−3.60	−21.28, 14.09	0.68

* *P*-value for independent samples *t*-test.

† Higher scores indicate best the quality of life.

‡ % PEF variability value of 100 indicates no variability, i.e. perfect control.

95% CI, 95% confidence interval; AQLQ, Asthma Quality of Life Questionnaire; DD, doubling dose; PEF, peak expiratory flow; PD20AMP (DD), provocation dose of adenosine monophosphate to reduce forced expiratory volume in 1 s by 20% in doubling doses; SD, standard deviation.

### Secondary outcomes

Juniper AQLQ and self-reported asthma symptom-free days and nights scores improved in both groups, more in the placebo group than the hookworm group (change in Juniper scores 15.34 vs. 10.66; symptom-free days 13.31% vs. 8.29%; symptom-free nights 12.63% vs. 9.71%), but neither this nor any other secondary outcome change differed significantly between groups (Table 3).

For both primary and secondary outcomes, there were no significant differences between the groups when we adjusted for potential confounders, carried out the sensitivity analyses or when outcomes were assessed using AUC (weeks 5–16) instead of change from the beginning to the end of the study (data not shown).

### Adverse effects

Localized skin redness and itching at the site of infection were reported in the hookworm group, particularly between 1 and 21 days after infection. Median daily scores of gastrointestinal symptoms were generally low but tended to be slightly higher in the hookworm than placebo group, significantly so for abdominal pain, and additionally for loss of appetite and nausea during the high-risk period (days 29–112; Table 4). There was no evidence to suggest a worsening of asthma during the first 4 weeks of the study. No clinically important change was observed in either of the groups in haemoglobin or serum albumen.

**Table 4:** Symptoms potentially attributable to hookworm infection experienced during the 16-week study period and high-risk period for participants with and without infection

	Daily score (scale 0–10) over total 16-week period				Daily score (scale 0–10) over high risk period‡			
Symptoms	Hookworm Median (range)	Placebo Median (range)	Difference in medians	*P*-value†	Hookworm Median (range)	Placebo Median (range)	Difference in medians	*P*-value†
Localized skin itching	0.28 (0.04, 2.17)	0 (0, 0.16)	0.28	<0.001*	1.40 (0.24,4.43)	0 (0, 0.67)	1.40	<0.001*
Localized skin redness	0.45 (0.06, 2.13)	0 (0, 0.23)	0.45	<0.001*	2.18 (0.33,4.19)	0 (0, 1.19)	2.18	<0.001*
Nausea	0.08 (0, 1.51)	0.01 (0, 0.46)	0.07	0.17	0.05 (0, 1.84)	0 (0, 0.61)	0.05	0.04*
Diarrhoea	0.06 (0, 2.03)	0.06 (0, 0.52)	0.00	0.69	0.04 (0, 1.90)	0.05 (0, 0.29)	−0.01	0.85
Abdominal pain	0.23 (0, 2.05)	0.03 (0, 1.04)	0.20	0.02*	0.30 (0, 2.59)	0.01 (0, 0.92)	0.29	0.02*
Flatulence	0.24 (0, 5.43)	0.12 (0, 1.65)	0.12	0.25	0.20 (0, 6.07)	0.05 (0, 1.77)	0.15	0.23
Indigestion	0.11 (0, 2.56)	0.01 (0, 0.43)	0.10	0.31	0.07 (0, 3.03)	0 (0, 0.67)	0.07	0.24
Loss of appetite	0.08 (0, 0.42)	0 (0, 1.18)	0.08	0.08	0.08 (0, 0.38)	0 (0, 0.83)	0.08	0.04*
Tiredness	0.18 (0, 4.47)	0.67 (0, 5.00)	−0.49	0.51	0.20 (0, 5.10)	0.36 (0, 5.52)	−0.16	0.52

‡ High-risk periods: localized skin symptoms (days 1–21), gastrointestinal symptoms and tiredness (days 29–112).

* *P*-value <0.05.

† *P*-value for Mann–Whitney-*U*-test.

### Confirmation of hookworm infection

**Fig. 2:**
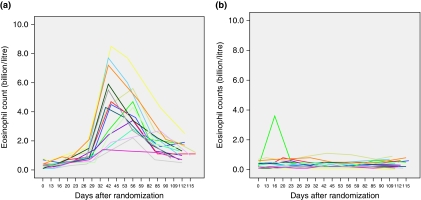
Peripheral blood eosinophil counts in participants randomized to (a) hookworm and (b) placebo.

All participants who received hookworm infection had a rise in peripheral blood eosinophil counts beginning around day 21 and peaking at 1.4–8.5 × 10^9^/L between days 42 and 84. Eosinophil counts in the hookworm group were all higher at the end of the study than at the time of infection (Fig. 2). Nine of the 16 participants who received hookworm and completed the study had eggs detected in their stool samples, appearing by week 6 in three and week 8 in six participants. At the final visit, stool cultures were positive for nine participants, eight of whom had had detectable eggs in their stool samples previously. All positive egg counts at this final visit were between 95 and 213 eggs/g feces.

### Assessment of participant blinding

At the end of the study, 11 participants in the hookworm group thought correctly that they had received infection (due to visible portals of entry on the skin and gastrointestinal disturbance); five did not know. Of those who received the placebo, eight correctly thought they had been allocated the placebo, two incorrectly thought they had received hookworm and six did not know. Thirteen out of 16 participants who received hookworm and completed the study elected to keep their infection.

## Discussion

This is the first reported intervention study of experimental hookworm infection in people with asthma. We found that the infection improved BHR by half a doubling dose more than the placebo group, although the confidence interval was wide and the difference between the groups was not significant. Our findings provide proof of concept that trials of experimental hookworm infection are feasible, well tolerated and, because the majority of participants chose to retain their infection after completion of the trial, that sustained infection is acceptable to patients.

The hypothesis tested in this study arises from multiple epidemiological studies demonstrating an inverse relation between helminth infection, especially hookworm, and both atopy and asthma [2, 3]. Previous intervention studies relevant to this hypothesis are limited to eradication studies, mostly performed in areas where hookworm was not the predominant endemic infection, and have produced mixed findings. Two studies found no difference in wheeze [16] or asthma [17] after antihelminth therapy when compared with controls. Conversely, another study reported a temporary improvement in asthma after albendazole, although this was not statistically compared with the placebo group [18]. Effects on atopy after antihelmith therapy are more consistent with three studies showing a clear increase in atopic response to allergen skin sensitization after treatment compared with controls [17, 19, 20] with a particularly strong effect for those with both hookworm and Ascaris infection [odds ratio (OR) 4.9, 95% CI: 1.48–16.19] [17]. However, other studies have found the effect of antihelminth treatment when compared with controls to be associated with a reduction in atopy [19] or to have no effect [16, 21].

A number of factors may explain why our trial did not demonstrate a statistically significant effect on BHR. It may be a false negative finding due to insufficient statistical power, particularly because the level of variability observed in our primary outcome, the change in PD_20_AMP, was greater than anticipated (SD 2.00 and 3.99 DD in hookworm and placebo groups, respectively). Blinding of treatment allocation was successful for those who received placebo and the clinical researcher who conducted the study visits and analysed the data, but less so for participants who received hookworm. However, we think it is unlikely that this appreciably affected our primary outcome, because this was based on objective measures of airway responsiveness. There is a possibility of random error in the PD_20_AMP values, and to reduce the impact of spurious results at randomization, we used the mean of the randomization and screening visits values to define the baseline PD_20_AMP, in accordance with our protocol. We did, however, observe a tendency for values to be higher at randomization than screening in both groups, which we believe is most likely due to a combination of improvement in inhaler technique and compliance with asthma treatment following education at the screening visit. However, we found no significant difference in change in PD_20_AMP between the groups regardless of whether values from the screening visit, randomization visit or the mean of the two was used as the baseline value. While people volunteering to take part in a study of experimental hookworm infection (or indeed any clinical trial) may not be representative of the asthma population in general, we have no reason to suspect that any such differences would interact with the clinical effect of hookworm infection.

Our previous studies showed 10 larvae to be effective in establishing infection to an intensity producing >50 eggs/g of feces [4, 6], but in the present study, eggs were not observed at any time in feces from several participants. Nevertheless, all infected participants exhibited marked elevation of peripheral blood eosinophilia, suggesting that those with no eggs in feces were infected with non-fecund or same-sex organisms. It is also plausible that the presence of allergic asthma reduces hookworm fecundity [22]. Another possibility is that the dose administered was not adequate to induce an immunological response of sufficient strength and type to be beneficial. We know from our study in people with allergic rhinoconjunctivitis that 10 infective larvae are sufficient to induce a natural immune phenotype with a weak indication of the establishment of an immune modulatory profile [7]. However, immune modulation may require further boosting to generate clinical benefit. Boosting could be achieved through the administration of repeated low-dose infections or through an increase in dosage. The former approach is probably more realistic, to avoid the undesirable adverse effects experienced with higher doses [4] and may mimic the pattern of natural and reportedly beneficial infection more closely. In addition, all participants in this study were receiving inhaled corticosteroid treatment, which may have inhibited the beneficial immunological shift induced by worms (such as the generation of regulatory T cell populations,) potentially conducive to therapeutic benefit and which has been linked to the alleviation of autoimmune disease in animal models and people with multiple sclerosis [23]. It is also possible that our study is negative because there is no true effect of hookworm infection in asthma, and that epidemiological associations observed previously have arisen from bias or confounding. However, given the consistency of the epidemiological evidence [3], we would propose that further studies of this hypothesis are both indicated and worth pursuing. Finally, the epidemiological findings may arise from reverse causation, whereby individuals with allergies are less likely to establish hookworm infection, but the high level of infection in our previous study, carried out in allergic individuals, argues against this [5].

In conclusion, our work at this juncture does not prove or refute the hypothesis that hookworm infection may protect against asthma, but establishes that trials of this intervention are feasible to pursue in clinical populations. Further studies of repeated low-dose infections, with longer periods of follow-up, and if possible, measures to ensure greater blinding of participants, are now indicated. Immunological monitoring for the presence of regulatory cell populations and cytokines and further assessment of the potential for polyclonal IgE to moderate allergic responses, should proceed in parallel, although recent field work suggests that the latter mechanism may not be operating in necatoriasis [24].
